# Supplementary material for: Early life bacteria and sibling exposure associate with restoration of the infant gut microbiome after cesarean section
Source: Nat Commun. 2026 Mar 30;17:4594. doi: 10.1038/s41467-026-71185-6 (PMC13194679; doi:10.1038/s41467-026-71185-6)
Supplement: Supplementary file 1 — Supplementary Information [file 41467_2026_71185_MOESM1_ESM.pdf]

Early life bacteria and sibling exposure associate with restoration of the infant gut microbiome after cesarean section

Jie Jiang[1,2], Casper Sahl Poulsen[1], Ulrika Boulund[1], Shiraz Shah[1], Urvish Trivedi[1,3], Madhumita Bhattacharyya[4], Avidan U. Neumann[4], Darlene L.Y. Dai[5], Charisse Petersen[5], Courtney Hoskinson[5,6], Theo J. Moraes[7], Piushkumar J. Mandhane[8,9], Elinor Simons[10], Meghan B. Azad[10,11], Padmaja Subbarao[7,12,13], Klaus Bønnelykke[1,14], Bo Chawes[1,14], Stuart E Turvey[5], Søren J. Sørensen*[3], Jonathan Thorsen*[1,14], Jakob Stokholm*[1,2,15]

[1]COPSAC, Copenhagen Prospective Studies on Asthma in Childhood, Copenhagen University Hospital - Herlev and Gentofte, Copenhagen, Denmark

[2]Department of Food Science, Faculty of Science, University of Copenhagen, Frederiksberg C, Denmark

[3]Department of Biology, Faculty of Science, University of Copenhagen, Copenhagen, Denmark

[4]Institute of Environmental Medicine and Integrative Health, Faculty of Medicine, University of Augsburg, Germany

[5]Department of Pediatrics, BC Children's Hospital, University of British Columbia; Vancouver, BC, Canada.

[6]Department of Microbiology and Immunology, University of British Columbia; Vancouver, British Columbia, Canada.

[7]Department of Pediatrics, The Hospital for Sick Children, Toronto, Canada

[8]Department of Pediatrics, University of Alberta, Edmonton, Canada

[9]Department of Medicine, Faculty of Medicine and Health Sciences, UCSI University, Kuala Lumpur, Malaysia

[10]Section of Allergy and Immunology, Department of Pediatrics and Child Health, University of Manitoba, Winnipeg, MB, Canada

[11]Manitoba Interdisciplinary Lactation Centre (MILC), Children's Hospital Research Institute of Manitoba, Winnipeg, MB, Canada

[12]Department of Medicine, McMaster University, Hamilton, ON, Canada

[13]Dalla Lana School of Public Health, University of Toronto, Toronto, Canada

[14]Department of Clinical Medicine, Faculty of Health and Medical Sciences, University of Copenhagen, Copenhagen, Denmark

[15]Department of Pediatrics, Slagelse Hospital, Slagelse, Denmark

*Joint senior authors

Corresponding author:

Professor Jakob Stokholm, MD, PhD
E-mail: stokholm@copsac.com
Website: www.copsac.com

Supplementary figures

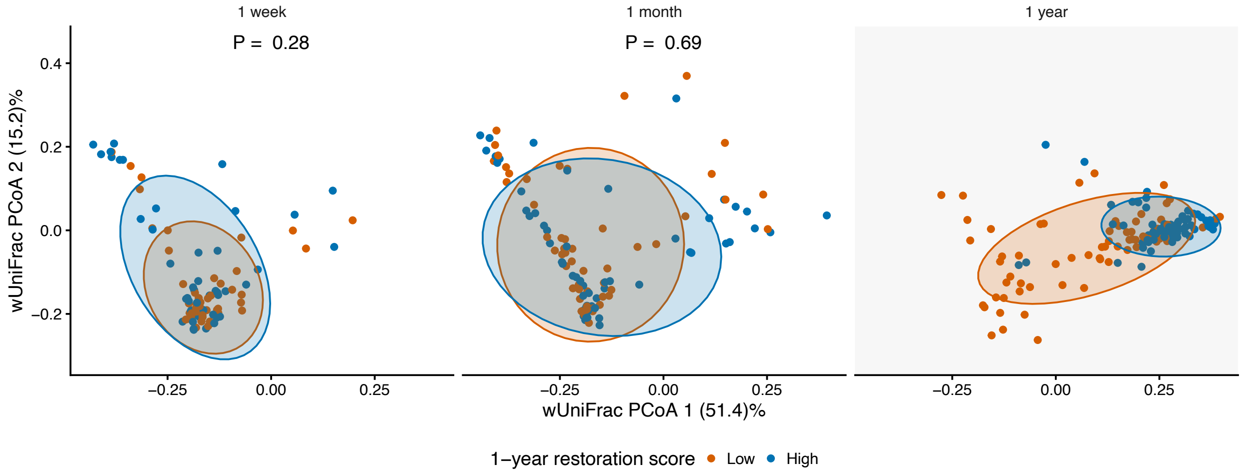

Supplementary Fig. 1. Comparison of beta diversity in fecal samples during the first year of life according to 1-year restoration score in the CS stratum. Principal coordinate analysis (PCoA) plots of wUniFranc distances for fecal microbial beta diversity in the first week, first month, and first year of life for children having high restoration score (orange, n = 41, 48, 53 at 1 week, 1 month and 1 year) and low restoration score(blue, n = 63, 68, 80 at 1 week, 1 month and 1 year). Gray shading at the 1-year time point marks when the restoration score was derived, hence groups are different by design. PERMANOVA was used to compare group differences, P values were computed by permutation. Each dot represents one fecal sample. Ellipses encompass 55% of the data points assuming a bivariate normal distribution.

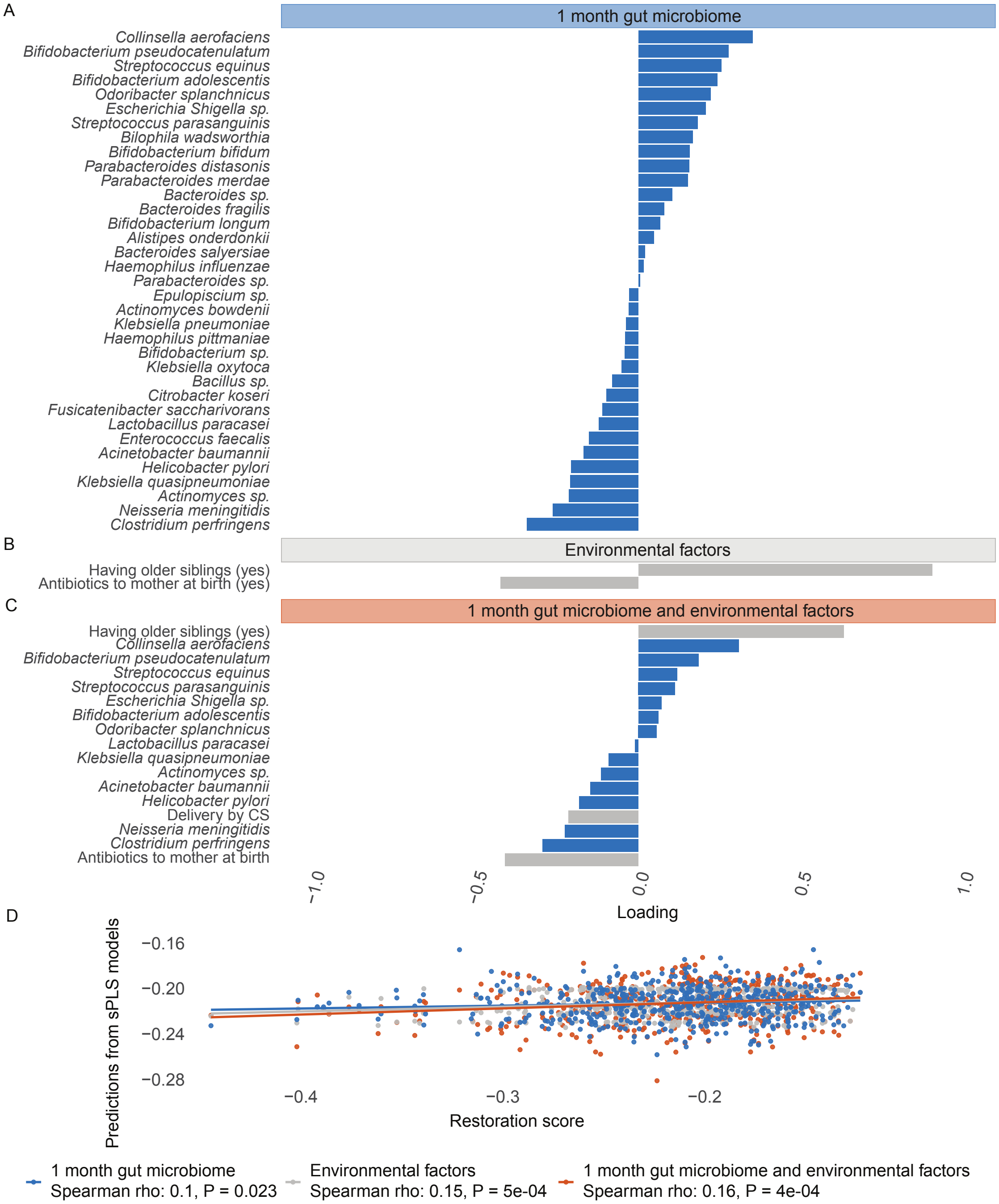

Supplementary Fig. 2. Sparse partial least squares (sPLS) models according to restoration score in the full cohort at 1 month of age. (A), (B), and (C) show loadings from models on gut microbiome at 1 month of age (152 species, 554 samples), environmental factors (39 factors, 512 samples), and combined factors (191 variables, 512 samples), respectively. Loadings represent the contribution of each variable to the sPLS models. Negative loadings indicate associations with lower restoration scores, while positive loadings indicate associations with higher restoration scores. (D) Spearman correlation between predictions from the above three sPLS models and the 1-year restoration score. The correlation coefficient and two-sided P value are shown in the legend. Blue for the sPLS model on gut microbiome, grey for the sPLS model on environmental factors and orange for the sPLS model on combined gut microbiome and environmental factors.

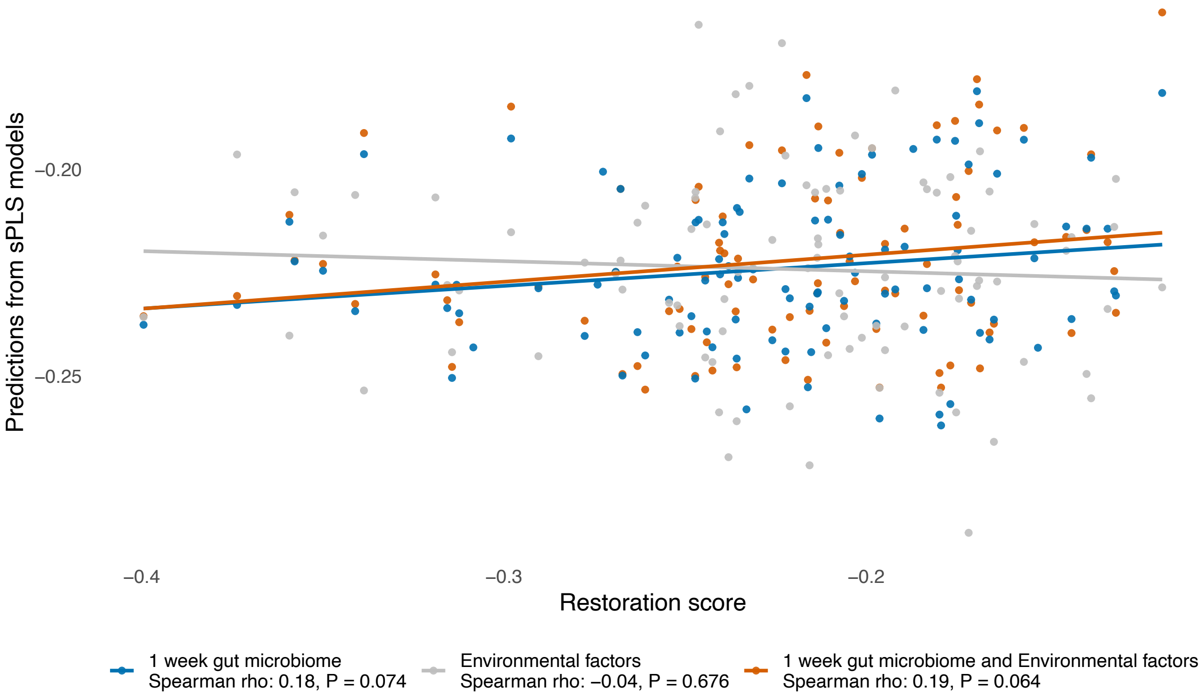

Supplementary Fig. 3. Spearman correlation between predictions from three sPLS models predicting 1-year restoration score in the CS stratum at 1 week. The correlation coefficient and two-sided P value are shown in the legend. Blue for the sPLS model on gut microbiome (140 species, 104 samples), grey for the sPLS model on environmental factor (36 factors, 93 samples) and orange for the sPLS model on combined gut microbiome and environmental factors (176 variables, 93 samples).

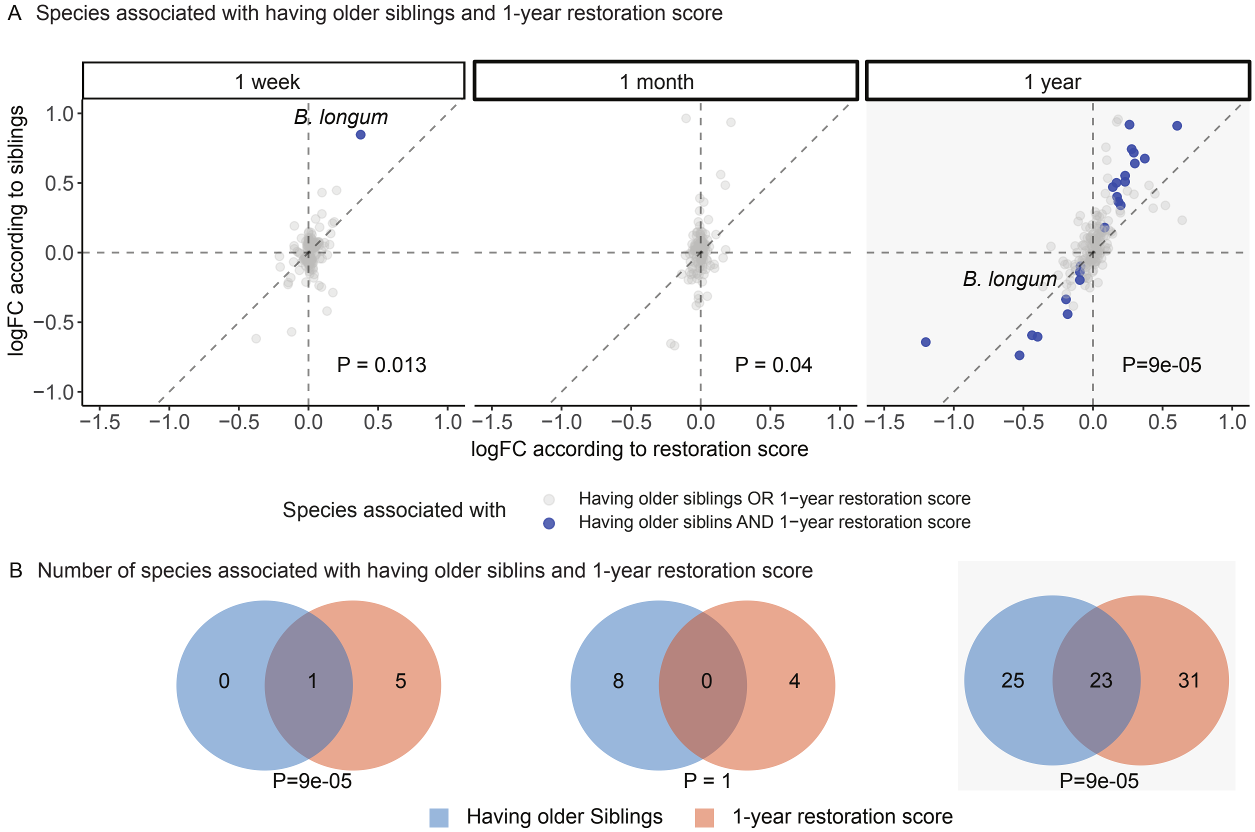

Supplementary Fig. 4. Differential abundance analysis reveals subset of species associated with having older siblings and restoration in the CS stratum. (A) Scatter plot comparing differential abundance analyses of having older siblings and the restoration score. Each point represents a species, those associated with both factors are highlighted in blue. Labeled species were significant at 1 week and 1 month. A positive log fold change value in the first quadrant (upper right) indicates an increase in the abundance of certain bacteria associated with a higher 1-year restoration score and having older siblings at home, while a negative fold change in the third quadrant (bottom left) indicates a decrease in the abundance of certain bacteria associated with a lower 1-year restoration score and not having older siblings. Permutation test P values (upper-tail) assess whether the concordance in direction between the two sets of associations is greater than expected by chance, a P value smaller than 0.05 indicates the concordant direction is unlikely under the null hypothesis. (B)Venn diagram indicating the number of differentially abundant species according to having older siblings and according to the 1-year restoration score. Permutation test P values (upper-tail) assess whether the observed overlap exceeds that expected by chance, a P value smaller than 0.05 indicates the overlap is unlikely under the null hypothesis.

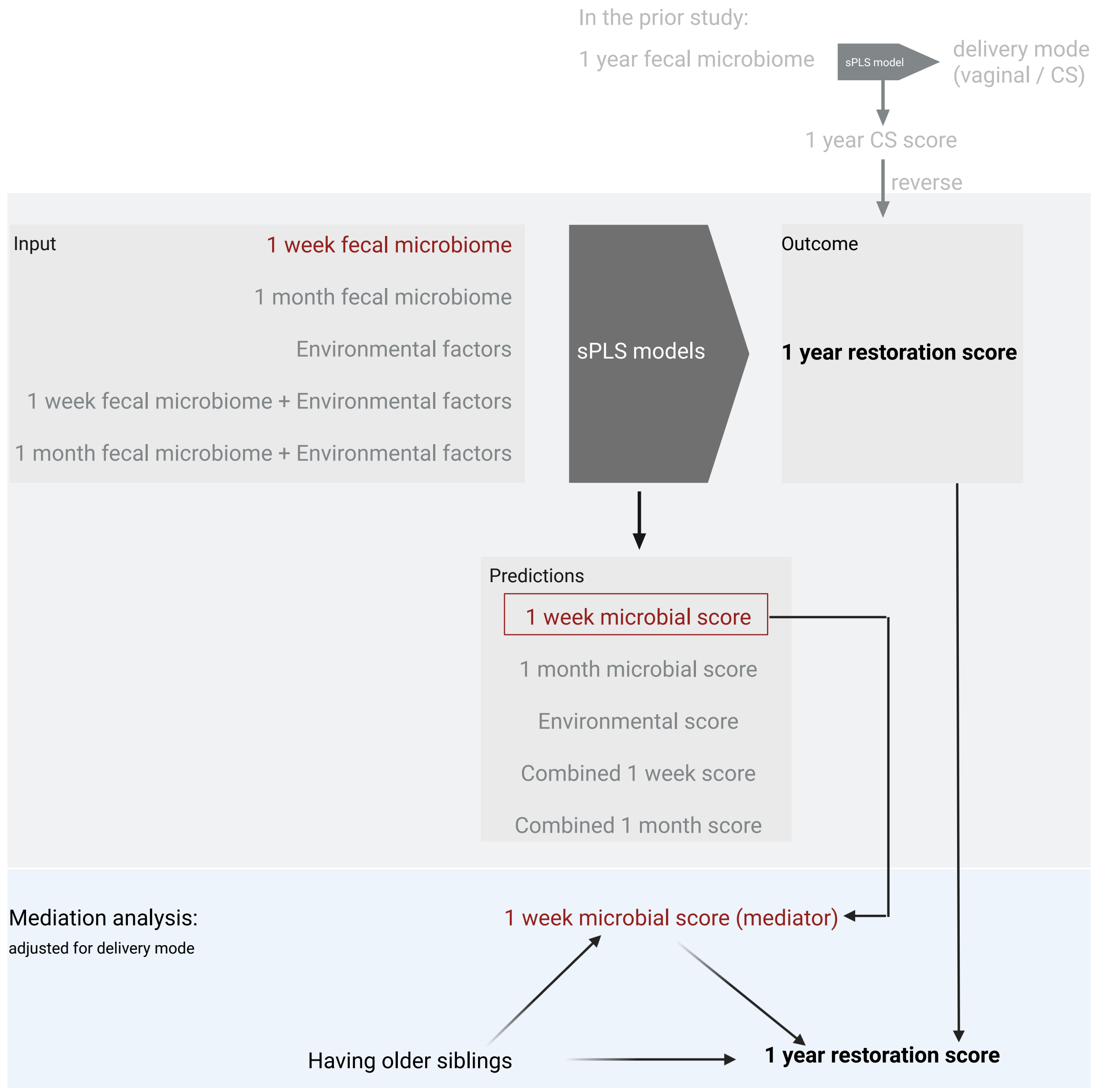

Supplementary Fig. 5. Flowchart illustrating the overview of the mediation analyses.

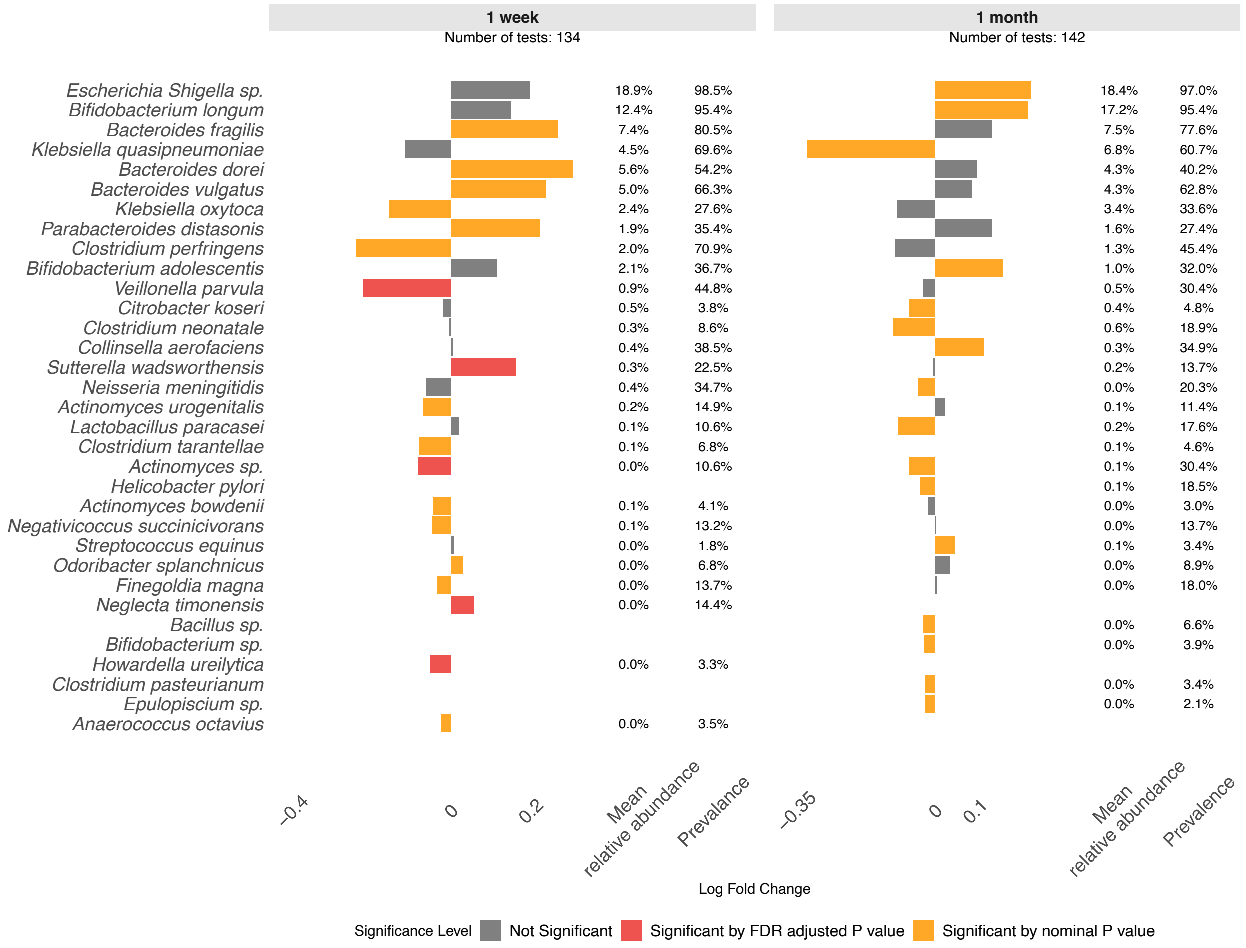

Supplementary Fig. 6 Differential abundance analysis on the species associated with 1-year restoration score in the vaginal stratum. Species with prevalence of at least 0.1% and relative abundance of more than 0.01% of the total were eligible. P values were calculated using two-sided tests. The species represented by the red bars were significant after FDR-adjustment, while species represented by yellow bars were nominally significant, and species represented by grey bars were not significant. A positive log fold change value indicates an increase in the abundance of the species as the 1-year restoration score increases, while a negative fold change indicates a decrease.
