# Supplementary material for: Early life bacteria and sibling exposure associate with restoration of the infant gut microbiome after cesarean section
Source: Nat Commun. 2026 Mar 30;17:4594. doi: 10.1038/s41467-026-71185-6 (PMC13194679; doi:10.1038/s41467-026-71185-6)
Supplement: Supplementary file 2 — Description of Additional Supplementary Files [file 41467_2026_71185_MOESM2_ESM.pdf]

File Name: Supplementary Data 1
Description: Baseline comparison of the two cohorts according to delivery mode. Associations between baseline variables and delivery mode were assessed by variable type. For continuous variables, the median and interquartile range (Q1, Q3) are shown, and P values were calculated using a two-sided Wilcoxon rank-sum test. For categorical variables, the proportion of each level within each delivery mode group is shown, and P values were calculated using Fisher's exact test when possible; otherwise, a chi-square test was applied.

File Name: Supplementary Data 2
Description: α-diversity according to 1-year restoration score in the full cohort and in the CS stratum. Mean and standard deviation (SD) of each α-diversity index are shown by visit and group. Estimates represent the difference in 1-year restoration score according to gut microbial α-diversity at 1 week and 1 month. LowCI and HighCI indicate the 95% confidence interval. P values were derived from two-sided linear models. Models in the full cohort were adjusted for delivery mode and sequencing depth, whereas models in the CS stratum were adjusted for sequencing depth.

File Name: Supplementary Data 3
Description: Environmental factors associated with 1-year restoration score in the full cohort and the CS stratum. Associations between environmental factors and the 1-year restoration score were evaluated separately in the full cohort and in the CS stratum using linear models. For each factor, the table shows the summary statistic (Stats), regression coefficient (Estimate), 95% confidence interval (LowCI and HighCI), nominal P value, and FDR adjusted P value. For continuous variables, the Estimate indicates the expected change in 1-year restoration score per 1-unit increase in the variable. For categorical variables, the Estimate indicates the expected difference in 1-year restoration score between the indicated category and the reference category. For continuous variables, Stats indicates the number of non-missing observations (N). For categorical variables, Stats indicates the proportion of participants in the indicated category.

File Name: Supplementary Data 4
Description: Differential abundance analysis (DAA) on the gut microbiome according to having older siblings or not in the full cohort at three time points. Reported values include log fold change (logFC), t statistic, nominal P value, and FDR-adjusted P value. Positive logFC values indicate higher abundance in children with older siblings, and negative logFC values indicate higher abundance in children without older siblings.

File Name: Supplementary Data 5
Description: DAA on the gut microbiome according to having older siblings or not in the CS stratum at three time points. Reported values include log fold change (logFC), t statistic, nominal P value, and FDR-adjusted P value. Positive logFC values indicate higher abundance in children with older siblings, and negative logFC values indicate higher abundance in children without older siblings.

File Name: Supplementary Data 6
Description: DAA on 1 year gut microbiome according to 1-year restoration score. Reported values include log fold change (logFC), t statistic, nominal P value, and FDR-adjusted P value. Positive logFC values indicate higher abundance in children with older siblings, and negative logFC values indicate higher abundance in children without older siblings.

File Name: Supplementary Data 7
Description: Environmental factors associated with 1-year restoration score in the vaginal stratum. Associations between environmental factors and the 1-year restoration score were evaluated separately in the vaginal stratum using linear models. For each factor, the table shows the summary statistic (Stats), regression coefficient (Estimate), 95% confidence interval (LowCI and HighCI), nominal P value, and FDR adjusted P value. For continuous variables, the Estimate indicates the expected change in 1-year restoration score per 1-unit increase in the variable. For categorical variables, the Estimate indicates the expected difference in 1-year restoration score between the indicated category and the reference category. For continuous variables, Stats indicates the number of non-missing observations (N). For categorical variables, Stats indicates the proportion of participants in the indicated category.

File Name: Supplementary Data 8
Description: Characteristecs of the CHILD cohort. Associations between covariates and asthma at 5 years of age were assessed by variable type. For continuous variables, the median and interquartile range (Q1, Q3) are shown, and P values were calculated using a two-sided Wilcoxon rank-sum test. For categorical variables, the proportion of each level within each delivery mode group is shown, and P values were calculated using Fisher's exact test when possible; otherwise, a chi-square test was applied.
