# Supplementary material for: Early life bacteria and sibling exposure associate with restoration of the infant gut microbiome after cesarean section
Source: Nat Commun. 2026 Mar 30;17:4594. doi: 10.1038/s41467-026-71185-6 (PMC13194679; doi:10.1038/s41467-026-71185-6)
Supplement: Supplementary file 4 — Reporting Summary [file 41467_2026_71185_MOESM4_ESM.pdf]

nature portfolio

Corresponding author(s): Jakob Stokholm

Last updated by author(s): Mar 1, 2026

Reporting Summary

Nature Portfolio wishes to improve the reproducibility of the work that we publish. This form provides structure for consistency and transparency in reporting. For further information on Nature Portfolio policies, see our Editorial Policies and the Editorial Policy Checklist.

Statistics

For all statistical analyses, confirm that the following items are present in the figure legend, table legend, main text, or Methods section.

n/a	Confirmed	
☐	☒	The exact sample size (*n*) for each experimental group/condition, given as a discrete number and unit of measurement
☒	☐	A statement on whether measurements were taken from distinct samples or whether the same sample was measured repeatedly
☐	☒	The statistical test(s) used AND whether they are one- or two-sided<br>*Only common tests should be described solely by name; describe more complex techniques in the Methods section.*
☐	☒	A description of all covariates tested
☐	☒	A description of any assumptions or corrections, such as tests of normality and adjustment for multiple comparisons
☐	☒	A full description of the statistical parameters including central tendency (e.g. means) or other basic estimates (e.g. regression coefficient) AND variation (e.g. standard deviation) or associated estimates of uncertainty (e.g. confidence intervals)
☐	☒	For null hypothesis testing, the test statistic (e.g. *F*, *t*, *r*) with confidence intervals, effect sizes, degrees of freedom and *P* value noted<br>*Give P values as exact values whenever suitable.*
☒	☐	For Bayesian analysis, information on the choice of priors and Markov chain Monte Carlo settings
☒	☐	For hierarchical and complex designs, identification of the appropriate level for tests and full reporting of outcomes
☐	☒	Estimates of effect sizes (e.g. Cohen's *d*, Pearson's *r*), indicating how they were calculated

Our web collection on statistics for biologists contains articles on many of the points above.

Software and code

Policy information about availability of computer code

Data collection	No software was used for data collection.
Data analysis	As described in methods, for data treatment and analysis, we used the open source statistical program R v4.4.0, predominantly the R-package phyloseq v1.48.0. Other R packages used include: picante v1.8.2, vegan v2.6-8, DAtest v2.8.0, mixOmics v6.28.0, tidyverse (version 2.0.0), mediation (version 4.5.1), Code is available on GitHub (https://github.com/finally-jay/restoration-score).<br>All P-values were two-sided and were considered statistically significant if the P-value was ≤0.05. When appropriate, we used FDR correction for controlling multiple testing.

For manuscripts utilizing custom algorithms or software that are central to the research but not yet described in published literature, software must be made available to editors and reviewers. We strongly encourage code deposition in a community repository (e.g. GitHub). See the Nature Portfolio guidelines for submitting code & software for further information.

Data

Policy information about availability of data

All manuscripts must include a data availability statement. This statement should provide the following information, where applicable:

- Accession codes, unique identifiers, or web links for publicly available datasets
- A description of any restrictions on data availability
- For clinical datasets or third party data, please ensure that the statement adheres to our policy

Individual-level personally identifiable clinical data from the children participating in the cohort cannot be made publicly available, to protect the privacy of the

participants and their families, in accordance with the Danish Data Protection Act and European Regulation 2016/679 of the European Parliament and of the Council (GDPR) that prohibit distribution even in pseudo-anonymized form. However, research collaborations are welcome, and data can be made available under a joint research collaboration by contacting the COPSAC Data Protection Officer (DPO), Ulrik Ralfkiaer, PhD (administration@dbac.dk). Requests will be answered within two weeks. Data use is restricted to purposes within childhood health and disease.
The accession numbers for the 16S rRNA gene sequence data of CHILD reported in this paper are BioProject accession (NCBI): PRJNA481046. Data described in the manuscript are available by registration to the CHILD database (https://childstudy.ca/childdb/) and the submission of a formal request. All reasonable requests will be accommodated. More information about data access for the CHILD Cohort Study can be found at https://childstudy.ca/for-researchers/data-access/. Researchers interested in collaborating on a project and accessing CHILD Cohort Study data should contact
child@mcmaster.ca.

Research involving human participants, their data, or biological material

Policy information about studies with human participants or human data. See also policy information about sex, gender (identity/presentation), and sexual orientation and race, ethnicity and racism.

Reporting on sex and gender

The aim of this study is not to find sex differences in the association of gut microbiome with the restoration status following cesarean section. Although information on sex was collected for the participants of both cohorts at birth in accordance with the ethics statement stated in the manuscript, and it was used as a covariate in the logistic regression to validate the association between restoration scores and asthma risk in both cohorts.
Summary statistics on the number of participants for each sex is reported previously (refer to DOI: 10.1126/scitranslmed.aax9929) and in Supplementary table 7.

Reporting on race, ethnicity, or other socially relevant groupings

Summary statistics on race (caucassian/non-caucassian) and other social relevant information (i.e., urbanicity, older sibling, pet ownership) are reported in supplementary table 1. All information was obtained by personal interview at clinical visits by medical doctors and research assistants both with paediatric training. Medical, familial, environmental, and socio-economic histories were assessed by predefined questions and closed response categories.
Refer to DOI: 10.1111/cea.12213 for further details on the COPSAC cohort characteristics.
Refer to DOI: 10.1136/thoraxjnl-2015-207246 for further details on the CHILD cohort characteristics.

Population characteristics

COPSAC2010 is a population-based mother-child cohort recruited in Denmark, with the overall aim of studying the mechanisms that lead to chronic diseases in childhood. The microbiome used in this study was extracted from faecal samples at 1 week, 1 month, and 1 year of age. In the analyses of assoicating early microbiome with 1-year restoration scores, the relevant covariate is delivery mode. And the technical variable (sequencing depth) was calibrated before sPLS modeling using linear regression. In the validation of assoicating resotration scores with asthma risk, the covariates included were gestational age, hospitalization after birth, antibiotics exposure to children at 1 year, having older siblings, family
asthma history, gender, race, birth season, (and study center for the CHILD study). CHILD study is a prospective longitudinal birth cohort study, which enrolled 3,405 subjects since pregnancy from 4 largely urban study centers across Canada (Vancouver, Edmonton, Winnipeg, and Toronto) from 2008 to 2012.The microbiome used in this study was extracted from faecal samples at 1 year of age.

Recruitment

COPSAC: Recruitment of 736 pregnant women was initiated late 2008 and ended July 2010 with 700 children. Refer to DOI:10.1111/cea.12213 for further details.
CHILD: Recruitment of 3624 pregnant women was initiated 2008 and ended 2012 with 3542 children. Refer to DOI: 10.1136/thoraxjnl-2015-207246 for further details.

Ethics oversight

COPSAC: The study was conducted in accordance with the guiding principles of the Declaration of Helsinki and was approved by the Local Ethics Committee (H-B-2008-093) and the Danish Data Protection Agency (2015-41-3696). Both parents gave oral and written informed consent before enrolment.
CHILD: Ethical approval for the CHILD Cohort Study, including the oversight of the CHILD biological samples and the CHILD database (CHILDdb), was obtained from the local Research Ethics Board of each study site: the University of British Columbia, the University of Alberta, the University of Manitoba, the Hospital for Sick Children and McMaster University.

Note that full information on the approval of the study protocol must also be provided in the manuscript.

Field-specific reporting

Please select the one below that is the best fit for your research. If you are not sure, read the appropriate sections before making your selection.

[x] Life sciences [ ] Behavioural & social sciences [ ] Ecological, evolutionary & environmental sciences

For a reference copy of the document with all sections, see nature.com/documents/nr-reporting-summary-flat.pdf

Life sciences study design

All studies must disclose on these points even when the disclosure is negative.

Sample size

COPSAC: All children with fecal samples collected and characterized by 16S rRNA sequencing (requiring at least 2000 reads) at any of the three time points of 1 week (n = 552), 1 month (n = 607), and 1 year (n = 625) were included in the analyses.
CHILD: All children with fecal samples collected and characterized by 16s rRNA sequencing at around 1 year visit (9 months to 12 months, n = 325) were included in the analyses. No power calculations were done before analyses, but this sample size had previously been sufficient to evaluate the microbiome vs asthma.

Data exclusions	Infants with missing information of delivery mode are excluded from the study.<br>Outcome:<br>COPSAC: Children with transient asthmatic phenotype (remission before age 6 years) are excluded.<br>CHILD: Children diagnosed as "possible asthma" are excluded.
Replication	We applied the sPLS model trained on the COPSAC2010 cohort on the CHILD cohort's 1-year samples (n=325) to create a restoration score. The restoration score, including association with delivery mode, older siblings and later asthma as well as early bacterial drivers, was successfully replicated in the independent Canadian birth cohort, CHILD.
Randomization	None, as this was an observational study. No microbiome intervention was done in the cohort.
Blinding	There was no control or placebo arm, so blinding was not applicable.

Reporting for specific materials, systems and methods

We require information from authors about some types of materials, experimental systems and methods used in many studies. Here, indicate whether each material, system or method listed is relevant to your study. If you are not sure if a list item applies to your research, read the appropriate section before selecting a response.

Materials & experimental systems

n/a	Involved in the study
☒	☐ Antibodies
☒	☐ Eukaryotic cell lines
☒	☐ Palaeontology and archaeology
☒	☐ Animals and other organisms
☒	☐ Clinical data
☒	☐ Dual use research of concern
☒	☐ Plants

Methods

n/a	Involved in the study
☒	☐ ChIP-seq
☒	☐ Flow cytometry
☒	☐ MRI-based neuroimaging

Plants

Seed stocks	Not applicable.
Novel plant genotypes	Not applicable.
Authentication	Not applicable.
